# Problematic Internet use and attitudes towards persons with disabilities – cross-sectional research among Polish students

**DOI:** 10.1186/s12909-023-04816-x

**Published:** 2023-12-04

**Authors:** Marta Kożybska, Iwona Radlińska, Arkadiusz Prajzner, Łukasz Krzywoszański, Beata Karakiewicz

**Affiliations:** 1 Pomeranian Medical University in Szczecin, Faculty of Health Sciences, Department of Social Medicine, Subdepartment of Medical Law, Ul. Żołnierska 48, Szczecin, 71-210 Poland; 2 Institute of Psychology, Faculty of Education and Psychology, University of the National Education Commission, Ul. Podchorążych 2, Krakow, 30-084 Poland; 3 Pomeranian Medical University in Szczecin, Faculty of Health Sciences, Department of Social Medicine, Subdepartment of Social Medicine and Public Health, Ul. Żołnierska 48, Szczecin, 71-210 Poland

**Keywords:** Internet addiction, Problematic Internet use, Disability, Attitudes, Students, Poland

## Abstract

**Background:**

Problematic Internet use (PIU) can have detrimental effects on physical, emotional, psychological, and social functioning. Besides well-described PIU correlations in psychological domains such as personality traits, or life satisfaction, and self-assessment, the social aspect of PIU risk also appeared to be important. This study aimed to investigate the association between PIU and attitudes towards persons with disabilities.

**Methods:**

A total of 595 Polish students aged 18–29 participated in this research by completing the Internet Addiction Test, Multidimensional Attitudes Scale Towards Persons With Disabilities, and personal information form.

**Results:**

The findings revealed that 30.6% of the respondents were at high risk for PIU. Moreover, heightened levels of PIU were more prevalent among male participants and students in technical fields of study than in those in medical and social fields of study. Increased PIU was also associated with more negative attitudes towards persons with disabilities in general and in the domains of emotions and behaviours. Additionally, prior personal contact with individuals with disabilities was related to both PIU rates and attitudes towards persons with disabilities in the domain of emotions and beliefs.

**Conclusions:**

This study highlights the prevalence of PIU among Polish students and emphasizes the need for preventive measures, particularly targeting male students and those in technical fields of study. The results indicate a relationship between PIU and attitudes towards individuals with disabilities. Further research is required to determine the direction of the relationship. It is recommended that educational programs provide opportunities for interaction with individuals with disabilities to promote understanding and acceptance.

## Background

The modern world requires using the Internet daily, in many aspects of our everyday life: working, studying, shopping, banking matters, making an appointment with a doctor, and many other situations. The Internet has become a tool that makes one’s life easier and, as it turned out during the COVID-19 pandemic, a tool that currently makes work and contact with the outside world possible, for instance, while in quarantine. However, the benefits of the Internet also pose threats: cybercrimes, cyberviolence, as well as, problematic Internet use (PIU). This notion implies using the Internet in a risky, excessive, or compulsive manner [1]. Such use has negative physical, emotional, psychological, and social consequences, including the occurrence of academic and occupational difficulties [1, 2].

This article aimed at verifying whether problematic internet use co-occurs with negative attitudes towards persons with disabilities. Research conducted so far has indicated that people with PIU have worse interpersonal relationships more frequently [3–5] and lower social skills [6] compared to those who use the Internet in an average manner. This may stem from the fact that those who have negative relationships with others may be more exposed to the development of PIU [3–5]. According to the research conducted by Romero-López et al., social skills such as conversation and social ease, empathic and positive feeling skills, and risk coping predict PIU. People characterized by social ease, emphatic people, and those who have positive feeling skills more frequently are less prone to problematic Internet use. Such people choose online social interactions less frequently and experience negative consequences in their social life caused by PIU less frequently [7]. It should also be noted that social anxiety symptoms occur more frequently in people with PIU than those without PIU [8–11]. Empathy may also play a crucial role in predicting problematic Internet use [7] and it is related to attitudes towards persons with disabilities. Some studies indicate a lower level of empathy among people with PIU [12, 13]. The study by Siyez has shown that students with a higher level of approval dependence and a lower level of empathy obtained more social benefits from the Internet, leading to excessive Internet use [13]. However, this relationship is not obvious. Interestingly enough, it has also been proved that the level of empathy is higher in those who spend more time on the Internet and have many close friends [6], which may be related to using the Internet to communicate with others. Besides well-described PIU correlations in psychological domains such as personality [14, 15], life satisfaction [16], and self-assessment [17], the social aspect of PIU risk also appeared to be important Interpersonal adaptation [18], relationship satisfaction [17], social security [19] and even emotional intelligence [20] have been identified as predictors of PIU among social factors. The causes and social consequences of PIU may be related to negative attitudes towards people with disabilities.

An attitude is a favourable or unfavourable response to someone or something, expressed in a belief, feeling, or intended behaviour. The cognitive component of an attitude is mainly manifested in categorising and stereotyping people. The affective component is an emotional way of interpreting the environment and leads to the realisation of the behavioural component, e.g. positive emotions can lead to altruism, and negative emotions can lead to prejudice and discrimination [21, 22]. Attitudes have their origins in internal dispositions, but above all in a person's social experiences, which are recognised as a leading factor [23].

The social learning theory proposed by Albert Bandura [24], later known as social cognitive theory, offers a compelling explanation of attitude formation. The important role of cognition in shaping both behaviours and attitudes is emphasized in this theoretical framework. According to this theory, the development of behaviour occurs through a modelling process, in which individuals acquire new behaviours through observation of others and their subsequent outcomes [24]. The social cognitive theory recognises that observed behaviours can be maintained or extinguished depending on the reinforcement received, thereby influencing an individual's attitude formation. By observing others and their social outcomes, behaviours are acquired, reinforced, or extinguished through a modelling process. Bandura highlights that this modelling process plays a fundamental role in shaping behaviours and, consequently, attitudes [24]. Furthermore, social cognitive theory introduces the concept of vicarious conditioning, wherein an individual's behaviour is influenced by observing the consequences experienced by others. This perspective highlights how modelling and vicarious conditioning can perpetuate both positive and negative patterns of behaviour. Positive reinforcement of certain behaviours encourages individuals to adopt similar attitudes and actions, while negative reinforcement or punishment discourages such behaviours. It is important to note that both desirable and undesirable attitudes can be acquired through this process [25].

The widespread use of the Internet has exposed young people to a variety of role models, some of whom may exhibit undesirable behaviour. As a result, socially negative attitudes may be reinforced by excessive exposure to peer-selected content. Problematic Internet use can be explained within the framework of social cognitive theory as a deficit in self-regulation skills. This deficit hinders the individual's ability to recognise the purpose of attitudes and to conform to socially acceptable norms. The social cognitive theory also addresses the development of pathological behaviours. Bandura claims that modelling and vicarious conditioning can reinforce pathological and undesirable behaviours, thereby perpetuating negative attitudes in society [24]. Individuals exposed to such behaviours may internalise and imitate them, leading to the spread of negative attitudes and actions. To fully understand the processes involved in attitude formation, social cognitive theory also considers additional factors such as self-evaluation and self-efficacy. Bandura explains that self-evaluation plays a crucial role in determining which attitudes and behaviours are considered acceptable and consistent with one's self-concept [26]. Meanwhile, self-efficacy refers to an individual's belief in their ability to perform certain actions successfully.

In today's society, the advent of the Internet has dramatically changed the availability of role models and sources of information. Young people in particular now have unprecedented access to a wide range of online role models, not all of whom demonstrate positive behaviours. The prevalence of peer-selected content, combined with the power of modelling and vicarious conditioning, can lead to the perpetuation of more socially negative attitudes [25]. In the context of social cognitive theory, PIU can be understood as a manifestation of poor self-regulatory skills. Larose et al. explain that individuals with lower self-regulation skills may have difficulty assessing the purpose of certain attitudes and may fail to align their behaviour with socially and internally acceptable norms [27]. This lack of self-regulation can lead to the development and reinforcement of undesirable and potentially harmful attitudes in the digital age.

In view of the multiple causes and consequences of PIU outlined above, its relationship with social attitudes can undoubtedly be anticipated. In particular, disturbances in interpersonal difficulties and limited social skills may lead to the reproduction of stereotypes (simplified, inflexible, and persistent social beliefs about a set of characteristics a priori attributed to a given object, e.g. a person with a disability is a victim of fate) and even prejudices (affective, irrational and unfair negative attitudes towards a specific social group, e.g. a person with a disability is a benefit defrauder) [28], which may lead to inappropriate social behaviour. In addition, it should be emphasised that negative attitudes towards people with disabilities have long been ingrained in societies [29]. The inaccurate social image of people with disabilities as victims of fate, as sad and unhappy people, with the stigma of mental or physical impairment, has led to social exclusion for many years [30–32].

Modulating these attitudes is difficult and continues to take place in various areas, including social advertising, the integration of persons with disabilities into society in education, work, and other fields, and the education of selected social groups. Raising the awareness of the whole society about persons with disabilities is one of the measures to ensure the protection of their rights and dignity by the Convention on the Rights of Persons with Disabilities of 13 December 2006 (Article 18) [33]. According to the Convention, disability is a concept that refers to persons with long-term physical, mental, intellectual, or sensory impairments who, when confronted with various environmental or mental barriers, are unable to participate fully in society. In this view, the essence of disability is not a health condition, but a barrier that causes marginalisation or exclusion [33]. Negative attitudes towards persons with disabilities are one of the key barriers to achieving equal participation of persons with disabilities in society.

Attitudes towards people with disabilities are rooted in the social environment [34]. This environment has largely moved online, especially during the COVID-19 pandemic. The prevalence of internet addiction has also increased during the pandemic [35, 36]. This significant change in the lifestyle of young people in recent years cannot be overlooked when looking for factors associated with attitudes towards persons with disabilities. Exploring the relationship between PIU and attitudes towards persons with disabilities may highlight the need for specific disability education activities for people exposed to PIU. This group of people includes young people, especially students, who are the largest recipients of Internet services in the world (75% of 15–24-year-olds use the Internet, compared to 65% of the rest of the population) [37]. As far as we know, the relationship between PIU and attitudes towards persons with disabilities has not been examined yet.

It should be mentioned that research on PIU and attitudes towards persons with disabilities is particularly crucial in the COVID-19 pandemic among young people, especially those who intend to work with the sick. The pandemic forced such people to learn remotely and reduce their interpersonal relations, whereas poor relations with teachers and students were considered to be a risk of developing PIU even before the pandemic [38]. Therefore, in our research, we decided to check whether PIU co-occurs with negative attitudes towards persons with physical disabilities among students representing various groups of fields of study.

Many researchers have attempted to determine which groups are most at risk of developing PIU. Previous research from Turkey [39], Norway [40], China [41], Germany [42], Greece [5], Korea [43], Hungary [44, 45], Mexico [46], Egypt [47], India [48], Iran [49], and finally the results of the 2022 meta-analysis [50] indicate that men are more likely to use the Internet in a problematic way. Fewer studies suggest that women are more likely to use the Internet in addictive ways [46, 51, 52]. Considering the purpose of Internet use, it appears that women are more likely to be addicted to social media and men to online games [53]. Since the general Internet Addiction Test was used in our research, we expect higher scores in men than in women.

The risk of PIU mainly affects young people. One of the most commonly studied groups is young adults, especially students [47, 48, 54–57].

Studies show that taking into account the field of study, the group least exposed to PIUs are students of medical faculties and the group most exposed are students of technical faculties [46, 58]. However, other researchers suggest that nursing students are more likely to become addicted to the Internet [59]. It should be emphasised that the above studies do not have a comparable number of representatives from different fields of study. Many studies on Internet addiction involve students from one type of study, usually medical [60–69]. Therefore, to determine the relationship between the field of study and PIU symptoms, there is a need for research that includes students from different fields of study.

Previous studies of attitudes with the MAS scale mostly involved students and showed that women had more positive attitudes in the cognitive and behavioural components, but more negative attitudes in the emotional component than men (e.g. [70, 71]). In addition, some studies have found no significant relationship between attitudes and gender using the MAS scale (e.g. [71–74]). Studies using other scales to measure attitudes (often the ATDP scale) that did not take into account the emotional component tended to show more negative attitudes among men (e.g. [75, 76]).

Other studies using different scales, in particular, different versions of the Attitudes Toward Disabled Persons (ATDP) questionnaire by Yuker et al. [77] measured the cognitive and behavioural domains of attitudes. A systematic review attempted to find differences in medical students' attitudes towards persons with physical disabilities [78]. It turned out that generally not the field of study itself is a significant predictor of attitudes (e.g. [22]), but above all, work experience with persons with disabilities acquired in the course of studies had an impact on increasing positive attitudes of students. The differentiation of attitudes occurred in the higher years of study [79, 80]. Social and medical faculties that provide contact with others, including persons with disabilities, as part of their placements ultimately increase positive attitudes towards persons with disabilities. An invariable predictor of posited attitudes for students in all majors was personal contact with people with disabilities outside of their studies [81].

The research focused on the level of problematic Internet use and negative attitudes towards persons with disabilities among Polish students. The gender of the participants, their fields of study, self-assessment of their health and financial situation, and whether they had previous personal contact with persons with disabilities were also taken into account as possible determinants of PIU and negative attitudes towards persons with disabilities.

As in most other PIU studies to date, the self-report questionnaire tool Internet Addiction Test (IAT) was used to assess it. The choice of the IAT instrument should provide an opportunity to directly compare the results of our research with studies using the IAT around the world, for example in the UK [82], USA, China, Korea, Turkey [83, 84], and India [85]. Despite the apparent changes in online functioning and attempts to separate the problems associated with this into the areas of smartphone use, social networking, or pornography, the meta-analyses continue to show the effective use of the IAT [50, 86, 87].

Having regard to the above, we distinguished the following research objectives: 1) to check the prevalence of PIU among Polish students; 2) to determine whether there is a relationship between PIU in students and their attitudes towards persons with disabilities; 3) to examine whether there are relationships between PIU and the attitudes towards persons with disabilities with gender, filed of study, knowing to disable person and self-assessment of financial and health situation.

Given the purpose and focus of the research as well as the theoretical premises presented, the following hypotheses were formulated:

1. The Internet Addiction Test (IAT) scores are associated with attitudes towards persons with disabilities.
2. Both genders differ in scores on the IAT and attitudes towards persons with disabilities.
3. Students having previous personal contact with persons with disabilities differ in scores on the IAT and attitudes towards persons with disabilities compared to students with no previous contact with persons with disabilities.
4. Subjects with various fields of study differ in scores on the IAT and attitudes towards persons with disabilities.
5. Self-assessment of one’s health is associated with scores on the IAT and attitudes towards persons with disabilities.
6. Self-assessment of one’s financial standing is associated with scores on the IAT and attitudes towards persons with disabilities.

## Methods

### Study procedure

The cross-sectional self-report online survey was conducted between December 2021 and the end of April 2022. The following criterion had to be met to participate in the survey: a Polish student, aged 18–29 with no disabilities. Students from all over Poland could complete a survey at panelariadna.pl or via Microsoft Forms. The targeted selection of respondents in the sample was used to guarantee a similar number of students representing various groups of fields of study: 1) natural science and engineering technology; 2) social science and humanities; 3) medical and health sciences; 4) law, economics, and management (Table 1). Before completing the survey, each student agreed to participate in the survey and was informed of the research objective and the possibility of withdrawing from the research at any stage.

**Table 1:** Responses to the questions on sociodemographic variables from personal information form

Variable	Value	*n*	*%*
Gender	Male	172	28.91
	Female	432	71.09
Place of residence	Countryside	137	23.03
	A small city with up to 100,000 inhabitants	170	28.57
	A big city with more than 100,000 inhabitants	288	48.40
Fields of study	Natural science and engineering technology	124	20.84
	Social science and humanities	135	22.69
	Medical and health science	211	35.46
	Law, economics, and management	125	21.01
To be in contact with a person with a disability	No	266	44.71
	Yes	329	55.29
Self-assessment of health situation	Very bad	12	2.02
	Bad	44	7.39
	Average	186	31.26
	Good	231	38.82
	Very good	122	20.50
Self-assessment of financial situation	Very bad	22	3.70
	Bad	64	10.76
	Average	255	42.86
	Good	190	31.93
	Very good	64	10.76

*n* number of observations

### Participants

The survey was completed by 669 subjects. By controlling for responses to the questions on age, student status, nationality, and disability, the final data analysis covered 595 respondents. Respondents above 29 years of age (*n* = 4), respondents not being students (*n* = 7), respondents not being Polish citizens (*n* = 7), and respondents who declared having disabilities (*n* = 56) were excluded from the initial database.

### Measures

#### The Internet Addiction Test (IAT)

Polish adaptation of the Internet Addiction Test (IAT) by Kimberly Young [88], which was developed by Ryszard Poprawa [89], was used to assess problematic Internet use. The Polish version of the IAT consists of 22 questions, which are answered on a scale from 0 to 5, where 0 is labelled “never” and 5 is labelled “always”. The IAT raw score is computed as the sum of the ratings of all items. One can score from 0 up to 110 points on the test. The higher the score, the higher the risk that a given person uses the Internet in a problematic manner. Furthermore, the score may be classified into the following categories: very low risk of Internet addiction (0–1 points for individuals of ≤ 24 years and 0 points for those of > 24 years), low risk of Internet addiction (2–10 points for individuals of ≤ 24 years and 1–6 points for those of > 24 years), moderate risk of Internet addiction (11–49 points for individuals of ≤ 24 years and 7–41 points for those of > 24 years), high risk of Internet addiction (50–79 points for individuals of ≤ 24 years and 42–75 points for those of > 24 years) and very high risk of Internet addiction (80–110 points for individuals of ≤ 24 years and 76–110 points for those of > 24 years). Cronbach's alpha is 0.94 [16]. Results from the last two ranges are considered to be alarming (problematic Internet use has occurred) [89]. To evaluate the validity, the author analysed correlations of many features measured by multiple methods and deemed the tool to be valid.

#### The Multidimensional Attitudes Scale Towards Persons With Disabilities (MAS)

It is a Polish adaptation of the Multidimensional Attitudes Scale Towards Persons With Disabilities by Findler et al. [90], which was developed by Radlińska et al. [91]. The MAS-PL scale is used to evaluate attitudes towards persons with disabilities. A respondent is asked to imagine various situations featuring a person in a wheelchair and to indicate the intensity of emotions (list of 16 emotions), thoughts (list of 10 thoughts), and potential behaviours (list of 8 behaviours), which may occur in a person without disabilities participating in such situations. The respondent answers on a scale from 1 to 5, where 1 is labelled “not at all” and 5 is labelled “very much”. Positive-worded items are reverse-scored. The higher the score, the higher the intensity of negative attitudes towards persons with disabilities. Apart from the overall score, the MAS-PL also provides subscales assessing three components: emotions, beliefs, and behaviours. The reliability of the overall result as well as these three components exceeds α = 0.80 [91].

#### Personal information form

Students were also asked questions on socio-demographic data (age, gender, field of study, year and level of study, nationality), the self-assessment of their financial situation, rated on a scale from 1 (very bad) to 5 (very good), self-assessment of their health situation, rated on a scale from 1 (very bad) to 5 (very good), and if the participant knew a person with disability personally and if the participant themselves were a person with a disability. All data obtained, including gender information, were data declared by students.

### Statistical analysis

Spearman's rank-order correlation was used to assess the relationships between raw scores on the Internet Addiction Test and attitudes towards persons with disabilities. The Mann–Whitney U-test with the rank-biserial correlation as a measure of the effect size was used to examine the gender differences in raw scores on the Internet Addiction Test and in negative attitudes towards persons with disabilities. The differences in raw scores on the Internet Addiction Test and negative attitudes towards persons with disabilities between subjects with or without previous personal contact with persons with disabilities were also tested using the Mann-Whitne U-test with the rank-biserial correlation as a measure of the effect size. The Kruskal–Wallis H test followed by Dunn’s test with p-value adjusted using Holm’s correction for multiple comparisons was used to examine differences in raw scores on the Internet Addiction Test and negative attitudes towards persons with disabilities and problematic Internet use persons with disabilities between students subjects in various fields of study. Statistical analyses were computed using the free and open-source statistical platform JASP [92], based on the R programming language for statistical computing [93]. The plots were prepared using the ggpubr R package [94].

## Results

The analysed sample consisted of 172 males (28.91%) and 423 females (71.09%). The students subject to analysis were aged 18–29 (*M* = 22.42; *SD* = 2.69). As can be seen in Table 1, nearly 50% of the students surveyed live in big cities with more than 100 100,000 inhabitants, whereas over 75% of the respondents, in total, live in towns and cities of different sizes. The number of students in natural science, social science and law, and economics fields of study is similar and ranges between 20 and 23%, while students in medical fields of study constitute over 35% of the sample. In addition, 43% and 59% of students claim that their financial and health situation is good and very good, respectively. On the other hand, 15% and 9% of students claim that their financial and health situation is bad and very bad, respectively. It can also be noticed that 55% of the students have personal contact with persons with disabilities.

The categorised IAT scores assessment indicated that 30% of students have a high and very high risk of Internet addiction. The frequencies for the risk levels of Internet addiction are presented in Table 2.

**Table 2:** Frequencies for the risk levels of Internet addiction

IAT scores		Risk levels of PIU	*n*	*%*	95% *CI*	
Age ≤ 24	Age > 24				*LL*	*UL*
0–1	0	Very low	11	1.85	1.00	3.30
2–10	1–6	Low	67	11.26	9.00	14.10
11–49	7–41	Moderate	337	56.64	52.60	60.60
50–79	42–75	High	157	26.39	23.00	30.10
80–110	76–110	Very high	23	3.87	2.60	5.70

*IAT* Internet Addiction Test,* n* number of observations, *CI* confidence interverbal, *LL* lower limit, *UP* upper limit

Very low and low risk of PIU was observed in 13% of students in total. Over half of the students had a moderate risk of addiction. The number of students who have been considered to be at high and very high risk of Internet addiction is 2.3 times higher than those at low and very low risk.

Table 3 shows Spearman’s correlations used to answer the research question on the relationships between problematic Internet use and attitudes towards persons with disabilities. Problematic Internet use was significantly correlated with the domain of emotions and behaviours as well as with the general attitudes towards persons with disabilities. The strength of these relationships was moderate, and their direction was positive—the high PIU was related to the higher level of negative attitudes towards persons with disabilities. Beliefs were the only domain of attitudes that were not correlated with problematic Internet use.

**Table 3:** Correlations between attitudes towards persons with disabilities and problematic internet use

Attitudes towards persons with disabilities [MAS scores]	Problematic Internet use [IAT scores]			
	*r*_*s*_	*p*	95% *CI*	
			*LL*	*UL*
Emotions	0.34	< 0.001	0.27	0.41
Beliefs	0.07	0.091	-0.01	0.15
Behaviours	0.30	< 0.001	0.22	0.37
Total	0.34	< 0.001	0.26	0.41

*Note: N* = 595

*IAT* Internet Addiction MAS—Multidimensional Attitudes Scale Towards Persons With Disabilities, Test, *r*_*s*_ – Spearman's rank correlation coefficient, *p* – significance, *CI* confidence interverbal, *LL* lower limit, *UP* upper limit

The Mann–Whitney *U* test was applied to assess the differences in raw scores on the Internet Addiction Test and negative attitudes towards persons with disabilities between males and females. The results are presented in Table 4.

**Table 4:** Differences between males and females in scores on the IAT and MAS

Variables tested		Males *N* = 174		Females *N* = 423		*z*	*p*	*r*_*rb*_	95%* CI*	
		*M*_*rank*_	*Me*	*M*_*rank*_	*Me*				*LL*	*UL*
Problematic Internet use [IAT scores]		351.27	41.00	276.34	27.00	4.820	< 0.001	0.25	0.15	0.35
Attitudes towards persons with disabilities [MAS scores]	Emotions	296.95	2.88	298.43	2.88	-0.095	0.924	0.00	-0.11	0.10
	Beliefs	321.37	2.40	288.50	2.30	2.114	0.034	0.11	0.01	0.21
	Behaviours	330.73	2.69	284.69	2.38	2.961	0.003	0.15	0.05	0.25
	Total	315.89	2.74	290.72	2.62	1.619	0.106	0.08	-0.02	0.19

*IAT* Internet Addiction Test, *MAS* Multidimensional Attitudes Scale Towards Persons With Disabilities, *N* number of observations, *M*_*rank*_ ranks of results, *Me* median, *z* the result of the Mann–Whitney test, *p* significance*, r*_*rb*_ rank-biserial correlation, *CI* confidence interverbal, *LL* lower limit, *UP* upper limit

**Fig. 1:**
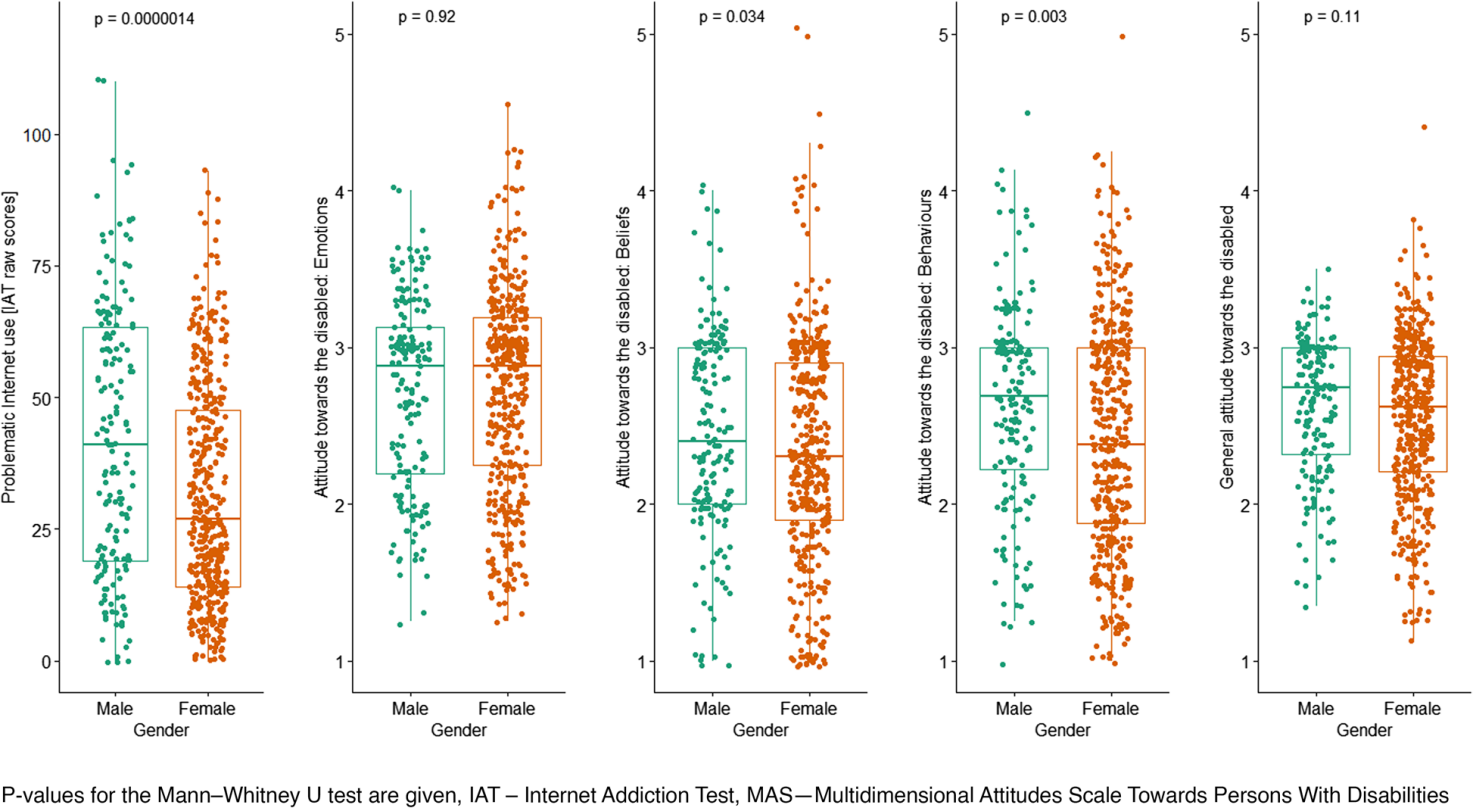
Differences in scores on the IAT and MAS between males and females

The analysis has shown that males scored significantly higher on IAT than females. The magnitude of this difference was small. Both genders did not differ significantly in negative attitudes towards persons with disabilities as well as the emotional domain of their attitudes. Interestingly enough, males had more negative attitudes than females in the domains of beliefs and behaviours. The magnitude of those differences was small. Scores on IAT and attitudes towards persons with disabilities in males and females are presented in Fig. 1.

The Mann–Whitney U test was used to examine the differences in raw scores on the Internet Addiction Test and negative attitudes towards persons with disabilities between subjects having or not having previous personal contact with persons with disabilities. The results are presented in Table 5.

**Table 5:** Differences in scores on the IAT and MAS and contact with a person with a disability

Variables tested		Knowing a person with a disability personally				*z*	*p*	*r*_*rb*_	95%* CI*	
		No *N* = 266		Yes *N* = 329						
		*M*_*rank*_	*Me*	*M*_*rank*_	*Me*				*LL*	*UL*
Problematic Internet use [IAT scores]		315.20	32.50	284.09	27.00	2.194	0.028	0.10	0.01	0.20
Attitudes towards persons with disabilities [MAS scores]	Emotions	318.72	2.94	281.24	2.75	2.644	0.008	0.13	0.03	0.22
	Beliefs	320.58	2.40	279.74	2.20	2.881	0.004	0.14	0.05	0.23
	Behaviours	310.53	2.63	287.87	2.50	1.599	0.110	0.08	0.02	0.17
	Total	321.77	2.72	278.78	2.59	3.033	0.002	0.15	0.05	0.23

*IAT* Internet Addiction Test, *MAS* Multidimensional Attitudes Scale Towards Persons With Disabilities, *N* number of observations, *M*_*rank*_ ranks of results, *Me* median, *z* the result of the Mann–Whitney test, *p* significance*, r*_*rb*_ rank-biserial correlation, *CI* confidence interverbal, *LL* lower limit, *UP* upper limit

**Fig. 2:**
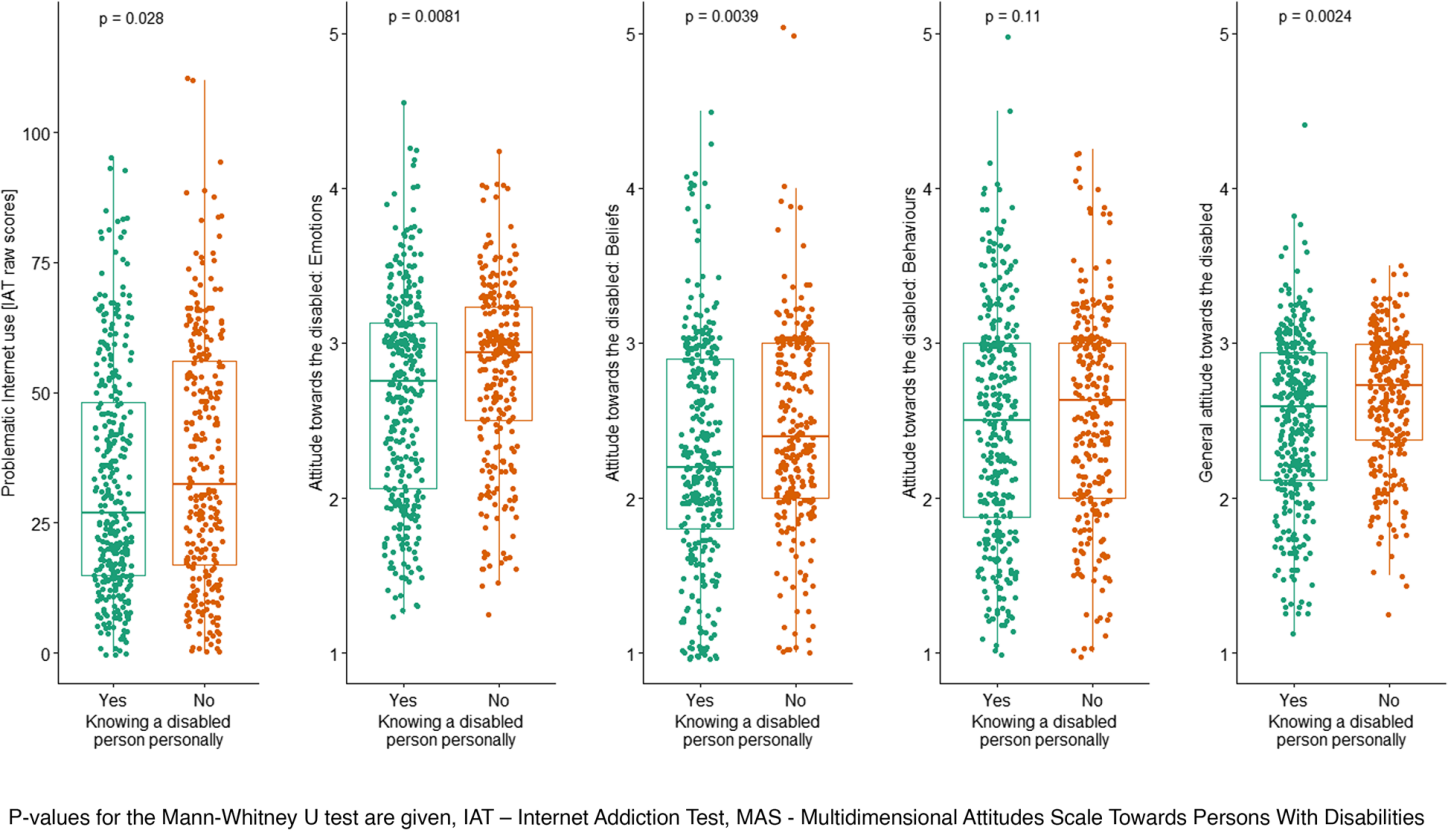
Differences in scores on the IAT and MAS and contact with a person with a disability

Subjects having previous personal contact with a person with a disability scored significantly lower on the IAT than those who did not know a person with a disability personally, but the magnitude of this difference was small. In addition, subjects having contact with a person with a disability have achieved significantly more positive general attitudes as well as the domains of emotions and fields towards persons with disabilities. The size of these effects was small. The behavioural component of attitudes did not differ significantly between those having and those not having personal contact with a person with a disability. Figure 2 showed differences in raw scores on the Internet Addiction Test and negative attitudes towards persons with disabilities between subjects with or without previous personal contact with persons with disabilities.

The Kruskal–Wallis *H* test was used to examine differences in raw scores on the Internet Addiction Test and negative attitudes towards persons with disabilities between students subjects in various fields of study. The results are presented in Table 6.

**Table 6:** Differences in scores on the IAT and MAS between subjects in various fields of study

Variables tested		Fields of study								*H*_*(3)*_	*p*	*ε*^*2*^	Post hoc
		NSET (1) *N* = 124		HSS (2) *N* = 135		MHS (3) *N* = 211		LEM (4) *N* = 125					
		*M*_*rank*_	*Me*	*M*_*rank*_	*Me*	*M*_*rank*_	*Me*	*M*_*rank*_	*Me*				
Problematic Internet use [IAT scores]		346.25	44.00	281.07	27.00	261.10	25.00	330.72	37.00	25.336	< 0.001	0.04	1 > 2,3 4 > 3
Attitudes towards persons with disabilities [MAS scores]	Emotions	313.51	2.94	327.26	3.00	250.15	2.56	331.79	3.00	26.150	< 0.001	0.04	1,2,4 > 3
	Beliefs	283.27	2.20	292.79	2.30	282.42	2.30	341.16	2.50	10.272	0.016	0.02	4 > 1,3
	Behaviours	319.18	2.63	305.57	2.63	255.84	2.25	339.97	2.75	22.378	< 0.001	0.04	1,2,4 > 3
	Total	306.68	2.65	321.44	2.76	248.98	2.44	346.82	2.79	30.080	< 0.001	0.05	1,2,4 > 3

*Note: Post-hoc analysis was performed by Dunn’s test with p-value adjusted using Holm’s correction for multiple comparisons*

*IAT* Internet Addiction Test, *MAS* Multidimensional Attitudes Scale Towards Persons With Disabilities, *NSET* Natural Science and Engineering Technology, *HSS* Social Science and Humanities, *MHS* medical and health science, *LEM* law, economics, and management; *N* number of observations, *M*_*rank*_ ranks of results, *Me* median, *H* the result of the Kruskal–Wallis test, *p* significance*, ε*^*2*^ ordinal epsilon-square

**Fig. 3:**
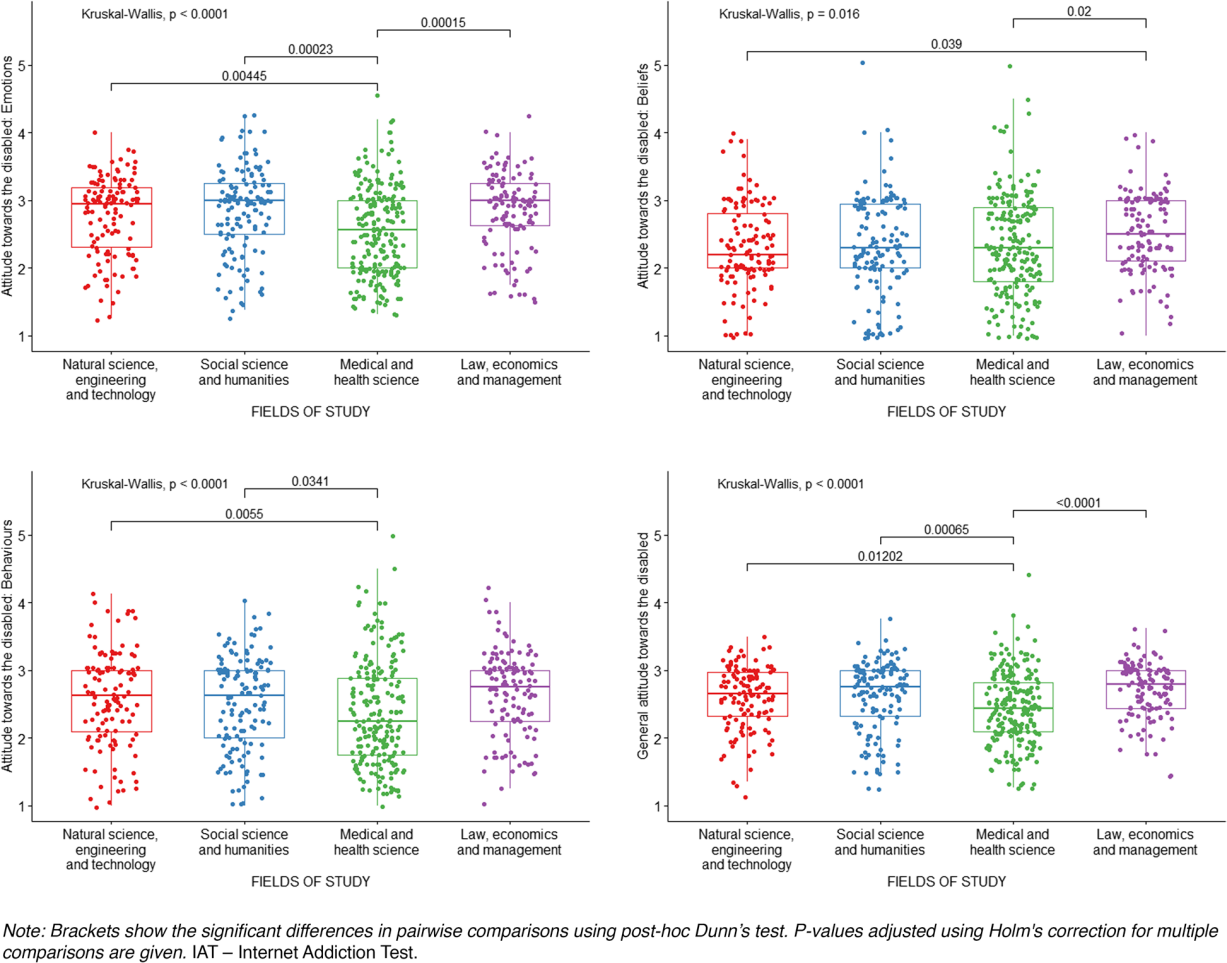
Differences in scores on the IAT between subjects with various fields of study

Students in various fields of study differed significantly in IAT scores, but the magnitude of these differences was small. The post-hoc analysis has shown that students in natural science, engineering, and technology studies had significantly higher scores of problematic Internet use than students in social and medical fields of study. The comparison also indicates that students in law, economics, and management fields of study had higher problematic Internet use than students in medical science. Differences in raw scores on the Internet Addiction Test between subjects with groups of students in various fields of study are shown in Fig. 3.

**Fig. 4:**
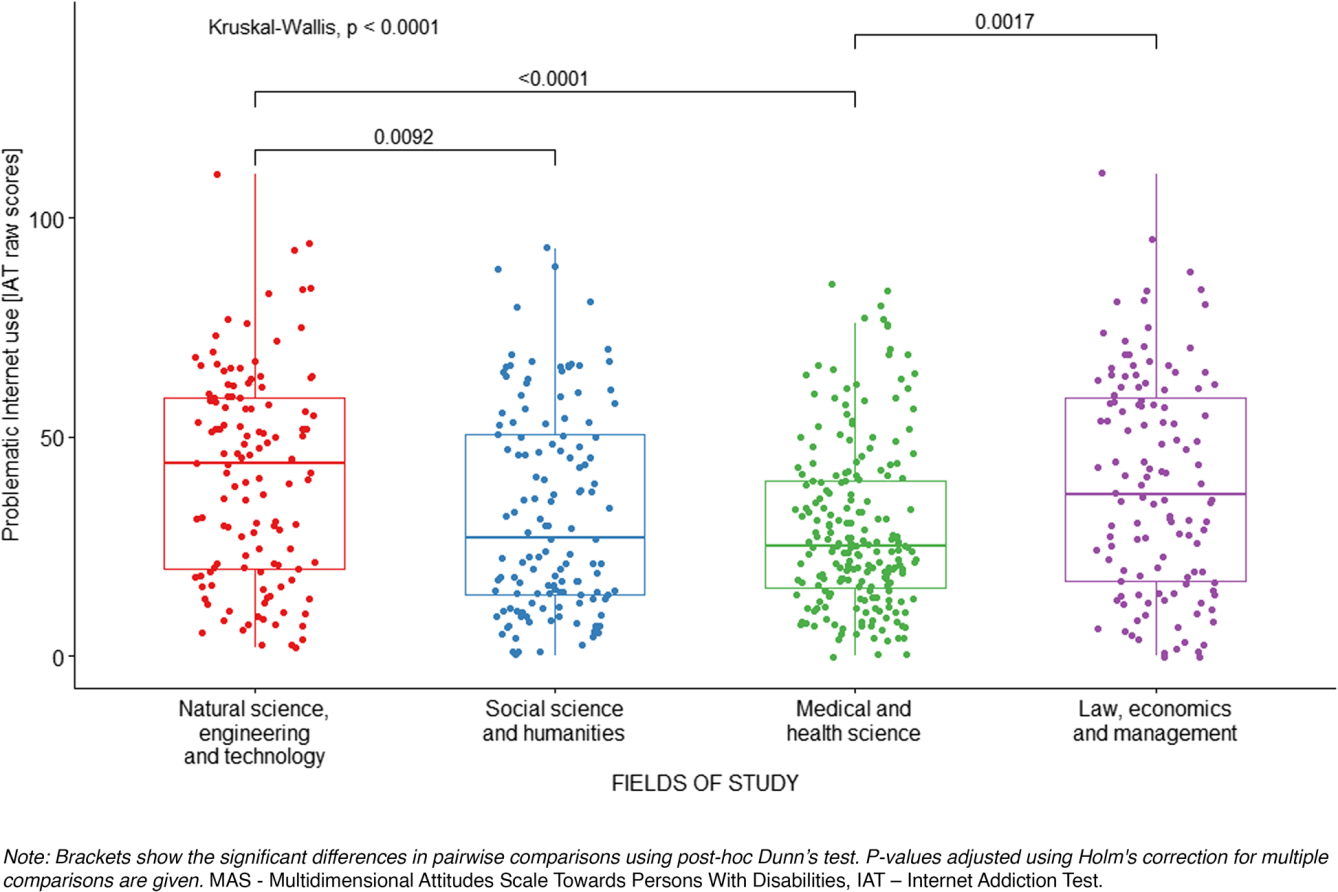
Differences in scores on the MAS between subjects with various fields of study

General attitudes towards persons with disabilities, as well as emotions, beliefs, and behaviours domain of attitude significantly differed among students in various fields of study. Effect sizes these differences were small. The post-hoc analysis has shown that students in medical fields of study had significantly more positive attitudes towards persons with disabilities in the domains of emotions and behaviours as well as the overall result than students in natural science, engineering and technology, social and humanities science, and law and economics fields of study. What is more, for both the overall result and the detailed domains, there are no differences in the level of attitudes towards persons with disabilities between students in natural, social sciences, and law and economics fields of study. For the domain of beliefs, the attitudes of medical and natural sciences engineering and technology students were significantly more positive in comparison to law and economics students. Differences in negative attitudes towards persons with disabilities between subjects with various fields of study were presented in Fig. 4.

Table 7 presents correlations between the variables tested and the self-assessment of health and financial situation using Spearman's rank correlation coefficient.

**Table 7:** Correlations of scores on the IAT and MAS with the self-assessment of health and financial situation

Variables tested		Health situation				Financial situation			
		***r***_***s***_	***p***	**95% *CI***		***r***_***s***_	***p***	**95% *CI***	
				***LL***	***UL***			***LL***	***UL***
Problematic Internet use [IAT scores]		-0.14	0.001	-0.22	-0.05	-0.23	< 0.001	-0.31	-0.15
Attitudes towards persons with disabilities [MAS scores]	Emotions	-0.07	0.077	-0.15	0.10	-0.17	< 0.001	-0.25	-0.09
	Beliefs	-0.11	0.006	-0.19	-0.03	-0.04	0.379	-0.12	0.05
	Behaviours	-0.04	0.391	-0.12	0.05	-0.11	0.008	-0.19	-0.03
	Total	-0.10	0.012	-0.19	-0.02	-0.14	0.001	-0.22	-0.06

*Note: N* = 595

*IAT* Internet Addiction Test, *MAS* Multidimensional Attitudes Scale Towards Persons With Disabilities, *r*_*s*_ Spearman's rank correlation coefficient, *p* significance, *CI* confidence interverbal, *LL* lower limit, *UP* upper limit

Correlations of scores on the Internet Addiction Test and self-assessment of health situation and financial situation were statistically significant. These relationships proved to be negative and weak. The higher the result of self-assessment of one’s health and financial situation the lower the problematic Internet use rate. Table 7 also presents significant relationships between the attitudes towards persons with disabilities and health situation and financial situation. These correlations were negative and weak. Interestingly enough, in the detailed areas of attitudes, beliefs were significantly correlated with health situation while emotions and behaviours were significantly correlated with the financial situation. A negative correlation has indicated that the higher the results of self-assessment of one’s health and financial situation, the weaker negative the attitudes towards persons with disabilities.

## Discussion

Problematic Internet use may cause difficulties in functioning in society, with attitudes towards the sick, the weak, or persons with disabilities being one of the aspects thereof. The research aimed at verifying whether PIU is related to attitudes towards persons with disabilities among Polish students.

First and foremost, it should be noted that PIU has proved to be a relatively common phenomenon in the group tested. The research has shown that nearly one-third of the students surveyed experienced problematic Internet use (high and very high addiction risk observed in 30.26% of respondents). Similarly, high scores were achieved by university students in Japan (38.2% of participants were classified as PIU) [95], and young adults in Bangladesh (27.1%) [96]. In other countries, lower scores were observed, for instance in the research conducted by Shi et al., 9.2% of Chinese medical students demonstrated moderate or severe levels (IAT ≥ 50) [97], in the research conducted by Di Carlo et al., 12.6% of the sample of Italian young adults had an IAT score of 50 or higher [98]. At the same time, as articles written by other authors indicate, the results of research within one population may differ considerably. In 2021, problematic Internet use was reported by 21% of Spanish university students in a study by Ramón-Arbués E. et al. [99], and 12.13% in a study by Aznar-Díaz et al. [46]. Since a considerable percentage of young people have symptoms of PIU, this phenomenon requires conducting research on its consequences and indicating a direction of preventative measures.

The present study has indicated that heightened PIU was related to more negative attitudes towards persons with disabilities in general and in the domain of emotions and behaviours. Beliefs were the only domain of attitudes that were not correlated with problematic Internet use. Therefore, the hypothesis on the relationship between PIU and attitudes towards persons with disabilities among Polish students regarding emotions and behaviours has been confirmed. Due to the cross-sectional nature of the research, it is impossible to answer the question about the direction of this relationship. On the one hand, negative attitudes towards persons with disabilities may reflect difficulties in social life caused by PIU. On the other hand, individuals with negative attitudes may be more exposed to the development of PIU. So far, other authors have not conducted research in this respect. Nevertheless, further research is required. Of particular interest may be the results of future research into the relationship between PIU and attitudes towards people with disabilities and the role of empathy and social skills in this relationship.

The present study has confirmed the hypothesis that students of different genders differ as to PIU. Men scored considerably higher on the IAT score than women. This is in line with the results of research conducted by other authors [5, 39–45, 47–50, 100, 101]. This tendency is also maintained in studies with a large sample and equal participation of women and men [102]. However, previous results do not confirm this pattern [103]. Therefore, the impact of gender on PIU is ambiguous, and the latest research shows that the purpose of using the Internet is more important [53].

The study has also confirmed that men and women did not differ significantly in attitudes towards persons with disabilities as well as the emotional area of their attitudes. Men had slightly more negative attitudes than women only in the domains of beliefs and behaviours. This corresponds to numerous results of research conducted by other authors [22, 71–73]. Particular attention should be paid to the emotional component that is identical for both genders (oftentimes negative) and the necessity of considering this aspect in research on attitudes. This requirement is met by the MAS-PL scale covering all 3 components of attitudes (emotions, beliefs, behaviours) used in the research.

As hypothesised, prior contact with persons with disabilities was significantly related to the areas subject [8]. However, these relationships were weak. Numerous studies, which were included in two systematic reviews, indicate that prior contact with persons with disabilities was related to more positive attitudes towards persons with disabilities in various population groups [78, 104]. The remaining elements confirming the hypothesis, in turn, could have been interpreted based on research on the social consequences of PIU [3–13].

The results have confirmed the hypothesis that students in various fields of study have different PIUs and different attitudes towards persons with disabilities. In all areas subject to research, students in medical fields of study achieved more desirable results. Natural sciences, engineering, and technology students achieved significantly higher PIU rates than students in social and medical fields of study. Law and economics students, in turn, achieved higher rates of Internet use than students in medical fields of study. In the research in question, medical and health science students had significantly more positive attitudes towards persons with disabilities in the domains of emotions and behaviours than natural sciences, engineering and technology, social and law, and economics sciences students. For the domain of beliefs, the attitudes of medical and natural sciences, engineering, and technology students were more positive than law and economics fields of study students. A recent study conducted on a Polish sample has failed to determine that the level of attitudes differs depending on the field of study [22]. Previous studies have also indicated that, in general, a field of study was not a considerable predictor of attitudes but rather the work experience gained during the course of studies in placements involving persons with disabilities [78]. However, such studies were conducted to a large extent using tools measuring a cognitive component (beliefs), e.g. ATDP Scale by Yuker et al. [77]. Therefore, the MAS scale showing a broader spectrum of attitudes (including emotions and behaviours) provides different results.

In the systematic review of 2021, Wang et al. [105] identified two main factors that influence public attitudes towards persons with different disabilities: people's knowledge about disability and the quality and frequency of their contact with persons with disabilities. Applying these findings to the results of our survey of student attitudes, it would seem that both education about disability and contact with persons with disabilities in the course of different fields of study are crucial in creating positive attitudes towards persons with disabilities. Furthermore, as reported by Case et al. in the 2021 meta-analysis [106], adapted physical activity service-learning has positive but small, effects on students' attitudes towards disability. According to the authors, several measures can be suggested to increase the impact of educational activities on attitudes towards people with disabilities in terms of the study programme and its implementation. In particular, programmes should include strategies specifically designed to change attitudes. Increase autonomy within service-learning by offering students a variety of choices and opportunities (e.g. different types of placements, locations, and disability groups to choose from). In addition, service-learning practitioners should promote optimal contact by encouraging the development of common goals and activities between students and persons with disabilities. It should be noted that professional contact with persons with disabilities does not only apply to students of medicine or health sciences. These persons are clients of many different industries and therefore positive attitudes towards them are expected from many different professionals.

The results have also confirmed the hypothesised relationships between a subjective self-assessment of one’s health and financial situation and PIU and the attitudes towards persons with disabilities among Polish students. The higher the result of self-assessment of one’s health and financial situation, the lower the PIU rate. Access to the Internet is becoming easier and wider for students and does not require large financial expenditure (wireless Internet on the campus, cheaper mobile Internet offers for students, possibility of earning on one’s own). Furthermore, undertaking studies implies achieving large freedom of decision-making related to entering an adult life (reducing or lack of parents’ control over Internet use or taking care of one’s health) [107]. As a consequence, the mechanism of PIU development is not based on little access to the Internet as it is not limited anymore, while larger financial capacities or better health are likely to increase the possibilities of spending free time away from home.

Significant relationships between the attitudes towards persons with disabilities and health situation and financial situation, respectively, have also been noted. In the detailed domains of attitudes, beliefs were significantly correlated with the declared health situation, while emotions and behaviours were significantly correlated with the declared financial situation. The higher the results of self-assessment of one’s health and financial situation, the more positive the attitudes towards persons with disabilities. No research has been conducted by other authors in this respect to date.

However, it should be noted that the self-assessment of one’s health and financial situation explained a maximum of 2% of differences in negative attitudes towards persons with disabilities as well as PIU.

## Conclusions

Problematic Internet use constitutes a serious problem among Polish students since it refers to nearly one-third of the respondents. A higher risk of PIU was observed in men than in women and among students of technical fields of study than those of medical and social fields of study. Moreover, the existence of relationships between PIU and attitudes towards persons with disabilities among Polish students regarding emotions and behaviours has been confirmed. Further research is required to determine the direction of this relationship. Previous personal contact with persons with disabilities was significantly associated with lower PIU rates. In addition, respondents who had previous personal contact with a person with a disability achieved significantly more positive attitudes towards persons with disabilities (in the domain of emotions and beliefs). However, both relationships were weak.

The conclusions of this study point to the need to implement some practical solutions. Firstly, due to the large number of people with PIU symptoms, an initiative aimed at preventing Internet addiction should be created for local health policy makers. Since our results revealed that men studying technical fields are more likely to demonstrate symptoms of PIU, further research is needed to determine which preventive methods are most effective in this group. The research to date suggests that this should include education about the risks associated with excessive use of the Internet, as well as the development of communication and social skills, taking into account the needs of people with disabilities, e.g. in the form of workshops during studies. Our research shows that ensuring contact with people with disabilities can help both to shape more positive attitudes and to reduce levels of PIU.

It appears that education about the risk of PIU as well as promoting and facilitating spending free time through contacts in a non-virtual world should become an essential element of education in all fields of study, particularly natural science, and engineering technology fields of study dominated by men. Encouraging women to choose technical fields of study may guarantee the interaction between both genders and contribute to decreasing tendencies towards PIU.

Activities to increase disability awareness and contact with persons with disabilities in different types of studies should be a key task. In particular, it should be made possible for persons with disabilities to participate in inclusive studies and activities in every field of study. Awareness-raising should include not only knowledge about disability and the problems of persons with disabilities but also the positive effects of contact with persons with disabilities (development of interpersonal skills, e.g. increased empathy). A variety of elective courses should also be introduced into the programme to enable students to come into contact with persons with different types of disabilities through internships and student placements. The tutors of such courses should actively help to establish positive relationships with persons with disabilities. It seems necessary to survey students' attitudes before and after the course on disability or contact with people with disabilities—consider modifying the course framework if the goal of improving attitudes is not achieved.

This study has confirmed significant relationships between the subjective self-assessment of one’s health and financial situation and PIU together with attitudes towards persons with disabilities. Having regard to the low clinical significance of the results of the present study, further analyses and research in this area are necessary. However, it appears that it may be crucial to provide students with financial support, particularly those in a difficult financial situation and gifted ones, through a scholarship and social aid system, which should be developed on an ongoing basis and enriched with offers of various fields of study.

## Data Availability

The data presented in this study is available on request from the corresponding author.

## Abbreviations

PIU: Problematic Internet Use
IAT: The Internet Addiction Test
MAS: The Multidimensional Attitudes Scale Towards Persons With Disabilities
MAS-PL: The polish adaptation of the Multidimensional Scale of Attitudes Towards People with Disabilities
